# Unmet Healthcare Needs, Catastrophic Health Expenditure, and Health in South Korea’s Universal Healthcare System: Progression Towards Improving Equity by NHI Type and Income Level

**DOI:** 10.3390/healthcare8040408

**Published:** 2020-10-16

**Authors:** Minsung Sohn, Xianhua Che, Hee-Jung Park

**Affiliations:** 1 Department of Health and Care Administration, The Cyber University of Korea, Seoul 03051, Korea; minsinge@cuk.edu; 2 Department of Health Policy Research, Daejeon Public Health Policy Institute, Daejeon 35015, Korea; chexianhua719@gmail.com; 3 Department of Dental Hygiene, College of Health Science, Kangwon National University, Gangwon-do 25945, Korea

**Keywords:** unmet healthcare needs, catastrophic health expenditure, health status, health insurance, health inequality, panel regression, medical aid, universal healthcare

## Abstract

This study examined the effects of healthcare inequality on personal health. It aimed to determine how health insurance type and income level influence catastrophic health expenditure and unmet healthcare needs among South Koreans. Unbalanced Korean Health Panel data from 2011 to 2015, including 33,374 adults, were used. A time-trend and panel regression analysis were performed. The first to identify changes in the main variables and, the second, mediating effects of unmet healthcare needs and catastrophic health expenditure on the relationship between health insurance type, income level, and health status. The independent variables were: high-, middle-, low-income employee insured, high-, middle-, low-income self-employed insured, and medical aid. The dependent variable was health status, and the mediators were unmet needs and catastrophic health expenditure. The medical aid beneficiaries and low-income self-employed insured groups demonstrated a higher probability of reporting poor health status than the high-income, insured group (15.6%, 2.2%, and 2.3%, respectively). Participants who experienced unmet healthcare needs or catastrophic health expenditure were 10.7% and 5.6% higher probability of reporting poor health, respectively (Sobel test: *p* < 0.001). National policy reforms could improve healthcare equality by integrating insurance premiums based on income among private-sector employees and self-employed individuals within the health insurance network.

## 1. Introduction

After Korea implemented universal health insurance for employees at large corporations in 1977, the range of beneficiaries expanded gradually, and universal health coverage for all was achieved in 1989 [1]. Health insurance beneficiaries can be classified into three groups, including (a) employees, for whom both the employer and employee each contribute to half of the insurance premium, and (b) self-employed workers, who pay their entire premium. In addition, approximately 3–4% of the population in the low-income bracket are (c) medical aid (MA) beneficiaries who are entitled to free insurance premiums and medical services. However, many poor families do not receive MA benefits. According to the national poverty standard, the bottom 7–8% of those in the low-income bracket live in poverty. Moreover, 15% of the population lives in relative poverty, meaning that they earn ≤50% of Korea’s median household income. The gap between those who receive MA benefits and those who live below the poverty line standard creates a significant blind spot in the current healthcare system. Therefore, despite universal health insurance that encompasses the entire population, the need for routine medical service coverage is increasing among the low-income bracket, which potentially contributes to a serious inequality in medical service usage [2].

In medical use, unmet healthcare needs may be incurred depending on health insurance type and income level. “Unmet healthcare needs” is a multidimensional concept that refers to the discrepancy between medical needs and accessibility [3]. It is defined as a failure to receive timely medical services needed to maintain optimum health, thereby increasing the risk of complications, declining health, and prolonged hospitalization [4]. The prevalence of self-reported unmet healthcare needs steadily decreased from 17% in 2013 to 11.5% in 2017 [5]. Nevertheless, it is 2–3 times higher than that of other Organization for Economic Co-operation and Development countries as reported by the European Union (EU) in 2016 [6]. The 2000 World Health Report published by the World Health Organization identified needs-based medical accessibility as a means of improving health [7]. Assessing needs-based medical accessibility is a key component in creating policies that efficiently distribute medical services [8] and could be a crucial index of the success of the universal healthcare system. Despite financial support through public health insurance, unmet healthcare needs continue to increase [9]. Analysis of the factors contributing to unmet healthcare needs has shown that income [10] and health insurance type [11] could exacerbate unmet need rates and health inequality.

Income inequality is intensified by catastrophic health expenditure, which refers to any health expenditure beyond a household’s financial capacity [12]. The percentage of families facing catastrophic health expenditure in Korea is ≥25% [13]. Low-income individuals are in the healthcare system’s blind spot and are likely to be exposed to catastrophic health expenditure. Especially, low-income families who do not receive MA benefits could face constant excessive health expenditures [14]; the rate of catastrophic health expenditure among low-income families is higher than that of other income brackets [15]. Exorbitant catastrophic health expenditure results in income loss and increased debts undermining a family’s overall quality of life [16,17]. Over time, this trend exacerbates health inequality. Based on their respective social status such as income, as well as the role of the health system in the Commission on Social Determinants of Health (CSDH) framework of the WHO, individuals experience differences in exposure and vulnerability to health-compromising conditions. Illness can reduce income and impact an individual’s social position, resulting in unmet healthcare needs or catastrophic health expenditure. The health system plays an important role in mediating the differential consequences of illness in people’s lives [18]. In Korea, there has been a consistent discussion of expanding health insurance coverage to protect families facing catastrophic health expenditure [19]. A recent health policy announced new support for health expenditures, including (a) out-of-pocket services up to KRW 20 million per year, (b) measures to prevent household bankruptcy resulting from catastrophic health expenditure among those in the bottom 50% of the low-income bracket, as well as (c) lowering the upper limit of personal health expenditure by income bracket.

Expanded insurance coverage could reduce catastrophic health expenditure and unmet healthcare needs, improving health outcomes [20,21]. In the past decade, Korea has implemented several policies to demonstrate its commitment to expand insurance coverage. In 2013, a policy was initiated to address the high health expenditures of four major diseases, namely, cancer, heart, cerebrovascular, and rare incurable diseases. Additionally, in 2017, Moon Care, which applied to previously uncovered services, was expanded in conjunction with the end of optional care, a deductible ceiling, and insurance coverage in advanced hospital wards and patient care. Nevertheless, expanded coverage did not reduce the financial burden of healthcare expenses [22], and the launch of Moon Care in 2017 resulted in a “balloon effect” (i.e., an increase in non-reimbursement that exceeds the coverage of benefits) that, recently, prompted criticism of coverage expansion.

Several Korean studies have reported varying estimates for catastrophic health expenditure and unmet healthcare needs according to insurance type. Catastrophic health expenditure for insured employees was higher than that for insured self-employed, and unmet healthcare needs were greater for MA beneficiaries than self-employed insured individuals [23]. Among health insurance subscribers, catastrophic health expenditure was more severe for insured employees than the self-employed insured, and the rate of unmet healthcare needs was higher for the self-employed insured than insured employees [24]. Recently, unmet healthcare needs among low-income families were reported to negatively influence health [10].

Despite persistent criticism of income and health inequality by health insurance types, efficient systematic change has not occurred owing to several factors: the financial burden or difficulty in prioritizing policy-making, discussions limited to MA beneficiaries, and a gap in coverage among vulnerable populations such as those in the second-lowest income bracket in Korea. A recent article by Park et al. [10] was the only study that examined how the interaction of unmet healthcare needs and income level affects health outcomes. However, the study did not take into account the form of the Korean health insurance system and excluded MA beneficiaries. In addition, previous research on health levels based on whether catastrophic health expenditure is incurred is insufficient. Therefore, this study aimed to investigate the extent of catastrophic health expenditure and unmet healthcare needs according to health insurance type and income level in Korea. The study also aimed to examine the relationship between unmet healthcare needs and catastrophic health expenditure, and their effect on health.

## 2. Materials and Methods

### 2.1. Data and Study Population

Data were sourced from the Korea Health Panel (KHP) survey administered by the Korea National Health Insurance Service and the Korea Institute for Health and Social Affairs. The KHP survey data provided information regarding medical utilization, medical expenditure, health status, and health behavior in Korea. The KHP survey is nationally representative of households in South Korea and conducted via computer-assisted personal interviews. Sampling is performed using a two-stage, stratified cluster sampling with probability proportionality. We used data from the sixth to tenth waves, which were conducted from 2011 to 2015. After excluding responses with missing values, we developed an unbalanced panel data, which ultimately included 33,374 cases involving individuals aged ≥18 years.

### 2.2. Variables

#### 2.2.1. Dependent Variables

The outcome variable was self-reported health. Catastrophic health expenditure and unmet healthcare needs are associated with a greater likelihood of poor health. The respondents who reported their health status as “poor” or “very poor” in response to the question “How do you think your health is usually?” were coded as 1. Those who reported it as “good,” “very good,” or “average” were coded as 0. Self-rated health is a useful measure in public health studies. There are also several studies that tried to test the validity and reliability of self-rated health measures and demonstrated that it was an effective indicator to predict death [25].

#### 2.2.2. Independent Variables

Health insurance type and income level were included as independent variables. Health insurance type was divided into three categories: (1) insured employees, (2) insured self-employed, and (3) MA beneficiaries. Equivalent income was divided into three categories: (1) high income, (2) middle income, and (3) low income. We then combined insurance type and income level, and categorized individuals into seven groups: high-income insured employees constituted the reference group and were coded as 0; middle-income insured employees were coded as 1; low-income insured employees were coded as 2; self-employed insured individuals who earned high, middle, and low incomes were code as 3, 4, and 5, respectively; MA beneficiaries were coded as 6.

#### 2.2.3. Mediation Variables

Unmet healthcare needs caused by economic hardship and catastrophic health expenditure were included as mediation variables. The respondents were asked, “Have you experienced an unmet need for healthcare during the past year?” Participants who responded “yes” were asked to choose their reason(s) from a list. Those who reported “economic burden” were identified as having experienced unmet healthcare needs owing to economic hardships in the preceding year. We categorized the outcome as “yes” if participants reported unmet healthcare needs or the inability to receive necessary medical interventions owing to economic burden, and “no” otherwise.

Catastrophic health expenditure is defined as the level of health expenditure relative to income, in line with Wagstaff and Doorslaer [26]. Total health expenditure includes pharmaceutical spending and the amount households pay to a medical institution for outpatient, inpatient, and emergency services. We set a threshold of 10% to define a catastrophic health expenditure episode. Respondents whose health expenditure was more than 10% of their household income were considered to have experienced catastrophic health expenditure and coded as 1, and others were coded as 0.

#### 2.2.4. Covariates

This study included individual- and family-level covariates. Individual-level variables included sex, age group, educational level, marital status, and employment status. Sex was dummy coded (male = 1; female = 2). Age was coded into three groups: 18–44 years, 45–64 years, and ≥65 years. Educational level was divided into three groups: respondents with a college or higher-level degree constituted the reference group and were coded as 1, high school graduates were coded as 2, and middle school graduates or those with a lower-level degree were coded as 3. Marital status was divided into three groups: the married group was treated as the reference group and coded as 1, divorced or widowed individuals were coded as 2, and single individuals were coded as 3. Employment status was categorized as follows: (1) permanent employment, (2) temporary employment, (3) self-employment, and (4) economically inactive. Respondents with chronic diseases were coded as 1, and those without chronic diseases were coded as 2. We included three family-level control variables: the number of family members, the proportion of economically inactive family members, and the proportion of family members with chronic diseases.

### 2.3. Statistical Analysis

Descriptive statistics and frequencies were calculated for all variables from 2011 to 2015. Chi-square analyses were performed for each predictor variable to identify factors associated with unmet healthcare needs, catastrophic health expenditure, and self-reported health. Total, direct, and indirect effects were estimated via panel probit regression following the guidelines described by Baron and Kenny [27]. Model I was created to assess the independent variables’ effects on the mediating variables (i.e., unmet healthcare needs and catastrophic health expenditure). Model II tested the independent variables’ effects on self-reported health. Model III included the mediating variables in Model II to test how changing the independent variables affected the dependent variables. We tested the significance of mediating effects via the Sobel test. All *p*-values were two-tailed with the significance level set at <0.05. All analyses were conducted using the statistical software STATA 15.

### 2.4. Ethics Approval Consent to Participate

This survey did not require formal ethical approval under national laws. We used only public data from the KHP, which did not include any personally identifiable data. Ethical and governance approvals were granted by the Korea Institute for Health and Social Affairs. All participants gave written informed consent for participation before they completed the survey.

## 3. Results

### 3.1. General Characteristics

Table 1 shows the general characteristics of the study population. We described general characteristics based on the preceding year’s results (2015). The study sample included high-income insured employees (19.5%), middle-income insured employees (43.6%), and low-income insured employees (8.2%). High-, middle-, and low-income individuals in the self-employed insured group comprised 4.4%, 17.1%, and 4.3% of the study sample, respectively. Furthermore, 2.8% of participants were MA beneficiaries. The proportion of women (58.0%) was higher than that of men (42.0%). Regarding educational levels, 37.3% of people reported at least a college-level education. In total, 24.9% of respondents were permanent workers, 21.2% were temporary workers, 19.0% were self-employed, and 34.9% were economically inactive. Of all respondents, 11.1% reported poor self-reported health, 3.6% experienced unmet healthcare needs, and 12.7% experienced a catastrophic health expenditure.

### 3.2. Characteristics According to Unmet Healthcare Needs, Catastrophic Health Expenditure, and Health Status

**Figure 1:**
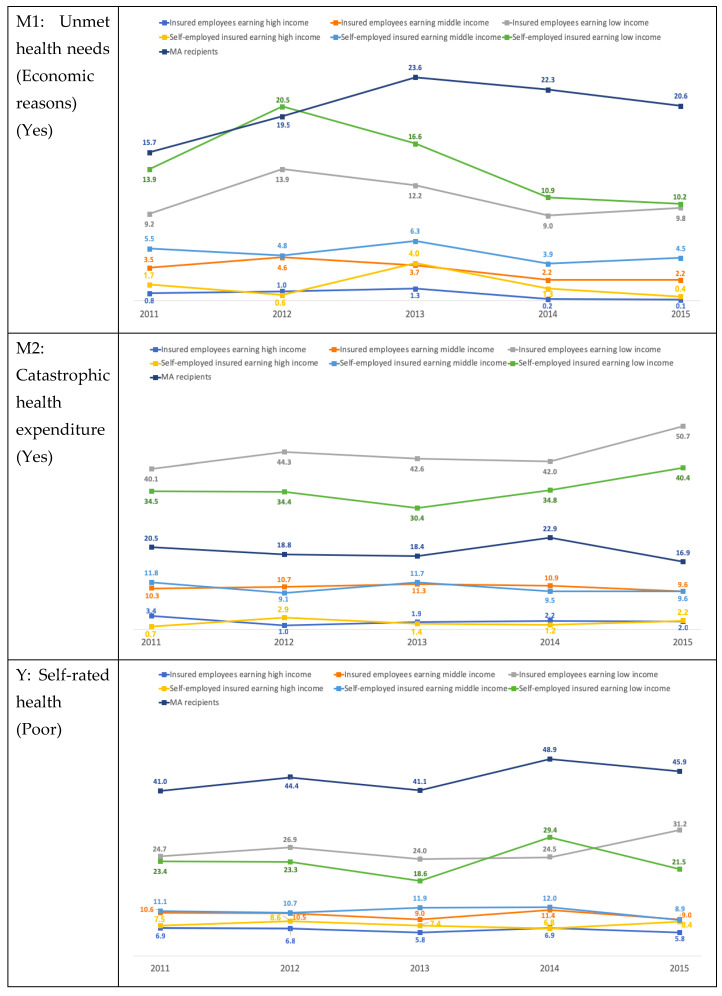
Proportion of participants reporting unmet healthcare needs, catastrophic health expenditure, and health status in South Korea from 2011 to 2015.

Figure 1 shows the trends in unmet healthcare needs, catastrophic health expenditure, and self-reported health from 2011 to 2015. The rate of unmet healthcare needs increased from 2011 to 2012 and declined thereafter. Additionally, MA beneficiaries, low-income insured employees, and low-income insured self-employed individuals showed high rates of unmet healthcare needs. MA beneficiaries showed a steady increase, with 20.6% reporting unmet healthcare needs in 2015.

Low-income insured employees and low-income insured self-employed individuals showed high rates of catastrophic health expenditure, which were higher than that of MA beneficiaries. In addition, 50.7% and 40.4% of low-income insured individuals reported catastrophic health expenditure in 2015. Moreover, these rates increased annually and at a rate higher than that of the high- or middle-income groups, or MA recipients.

In the case of health status, the low-income groups were more likely to report poor health status relative to those with higher incomes. Poor health in 2015 was highest among MA beneficiaries (45.9%), followed by low-income insured employees (31.2%), and low-income insured self-employed individuals (21.5%). The high- and middle-income groups showed stable rates of poor health status from 2011 to 2015, but the low-income and MA groups showed increasing trends during this period.

Table 2 shows the results of the chi-squared tests. In total, 44.4%, 26.4%, and 23.6% of MA beneficiaries, low-income insured employees, and low-income insured self-employed individuals reported poor health status, respectively. In addition, 20.2%, 10.7%, and 14.2% of MA beneficiaries, low-income insured employees, and low-income insured self-employed individuals reported unmet healthcare needs, respectively. Further, 44.1% of low-income insured employees and 35.2% of low-income insured and self-employed individuals experienced catastrophic health expenditure, and these proportions were high relative to MA beneficiaries (19.5%). Women aged 65 years or older who reported lower educational levels, lived without partners, and had been diagnosed with chronic diseases were significantly more likely to report poor health status, unmet healthcare needs, and a catastrophic health expenditure relative to other participants. 

### 3.3. Effects of Catastrophic Health Expenditure and Unmet Healthcare Needs on Health Status According to Health Insurance Type and Income Level

As shown in Table 3, after controlling for individuals’ demographic and family characteristics, low-income insured employees had a 6.2% higher probability of reporting unmet healthcare needs and low-income insured self-employed individuals show a 10.0% higher probability of doing so than high-income insured employees. The MA beneficiaries were 12.2% more likely to report unmet healthcare needs relative to the reference groups. The effect of the independent variable on the unmet healthcare needs (as a mediating variable) was statistically significant.

Regarding catastrophic health expenditure, low-income insured employees and low-income insured, self-employed individuals had a 27.0% and 25.4%, respectively, higher probability of reporting a catastrophic health expenditure relative to high-income insured employees. The MA beneficiaries (8.1%) experienced a higher probability of reporting a catastrophic health expenditure relative to the comparison groups. The independent variable’s effect on catastrophic health expenditure (as a mediating variable) was statistically significant.

Model II showed significant direct effects of the independent variable on self-reported health. Low-income insured employees and low-income insured self-employed individuals reported a 4.5% and 5.0% higher probability of poor health status, respectively, than the higher-income groups. The MA group reported a 17.2% higher probability of poor health status.

In the final model, low-income insured employees reported a 2.2% higher probability of poor health status while low-income insured self-employed individuals had a 2.4% higher probability of reporting poor health. The MA beneficiaries had a 15.6% higher probability of reporting poor health relative to those who earned higher incomes. Furthermore, individuals who reported unmet healthcare needs were 10.7% more likely to report poor health. Individuals who experienced a catastrophic health expenditure reported 5.6% higher probability of poor health status relative to those who did not report catastrophic health expenditure. The result of the Sobel test showed *p* < 0.001 for low-income insured employees; low-income insured self-employed individuals; MA beneficiaries. These results indicate that poor health was more common among low-income national health insurance (NHI) recipients and MA beneficiaries, and it was mediated by catastrophic health expenditure and unmet healthcare needs.

## 4. Discussion

This study aimed to examine the effect of catastrophic health expenditure and unmet healthcare needs on South Koreans according to health insurance type and income level, based on medical panel data for 5 years, from 2011 to 2015. The study also examined how catastrophic health expenditure and unmet healthcare needs affected health outcomes, which, to our knowledge, has not been examined extensively in the literature. The results of this study could contribute to national policy measures to resolve catastrophic health expenditure and the unmet healthcare needs in Korea.

The results show the effects of health insurance type and income level on the unmet healthcare needs of participants. The unmet healthcare needs reported by MA beneficiaries, low-income self-employed, and low-income insured employees were approximately 12.2%, 10.0%, and 6.2% higher, respectively, than that reported by high-income insured employees. This suggests that, in Korea, the risk of experiencing an unmet healthcare need is higher among MA beneficiaries, relative to those with insurance policies [23], and among those with insurance, the rate of unmet healthcare needs among self-employed individuals was higher than that among insured employees [24]. In other words, the population of individuals vulnerable to unmet healthcare needs included MA beneficiaries and low-income insured self-employed individuals. Of note, MA beneficiaries persistently experienced unmet healthcare needs, despite a lower risk of catastrophic health expenditure relative to other groups, because they did not have to make deductible payments, which forced them to limit medical services availed because of the deduction ceiling. It has also been confirmed that the lowest income and self-deployed benefits are more likely to experience unmet dental needs in the dental field of Korea [28]. Recently, Moon Care allowed for increased insurance coverage aimed to mitigate catastrophic health expenditure and unmet healthcare needs. However, this scheme has faced criticism because it is still too limited [29]; a high proportion of MA beneficiaries are elderly with poor health and limited or insufficient medical service access.

Another noteworthy finding was that the rate of unmet healthcare needs in low-income insured self-employed individuals was as high as that of MA beneficiaries. In Korea, the self-employed group has a particularly large number of low-income people, including small business owners. Unlike insured employees, they cannot rest even if they are sick because they are not guaranteed to have paid sick leave; thus, treatment is often delayed. Therefore, it is essential for Korea to improve medical accessibility for MA beneficiaries and for those in the healthcare coverage blind spot. The poorest strata and the second-lowest income bracket are not adequately protected by the national policies. It is necessary to review these policies and consider approaches such as further segmentation of low-income groups according to income levels. In addition, the higher incidence of unmet healthcare needs in low-income self-employed insured compared to low-income employee insured, even if the same level of insurance premiums is low, indicates there is inequality by the type of insurance in the NHI system. Addressing this inequality might contribute towards resolving the burden of health expenditure and improving accessibility for the poorest strata of society in terms of health insurance and MA. Further, it is essential to consider the introduction of the Sickness Benefit system in South Korea. This system is not limited to the health insurance system, such as contribution of the health insurance premium, but preserves the loss of income of vulnerable workers such as self-employed people.

The mediation analysis of unmet healthcare needs indicates that overall health deteriorated among MA beneficiaries and the low-income insured, self-employed individuals. This is consistent with the findings of a domestic study in which the unmet needs for healthcare in a low-income group exerted a negative impact on health [10]. As an example, China introduced the Critical Illness Insurance System in 2012 to improve health equality by reducing patients’ burden of health expenditure and allowing timely treatment, which led to both short- and long-term health improvement [21]. Moreover, after China launched the New Cooperative Medical Scheme system in 2003, and expanded insurance coverage in 2009, inequality in catastrophic health expenditure declined [30].

The mediation analysis of catastrophic health expenditure showed that such expenditure was higher in low-income insured employees and low-income self-employed individuals than the MA beneficiaries. In a previous Korean study, the highest rate of catastrophic health expenditure was observed in insured employees [24]. Considering type of health insurance, there is a higher probability of catastrophic health expenditure among insured employees who maintain a relatively stable income compared to self-employed individuals. The time-series trend analysis suggested that a continued increase in catastrophic health expenditure is likely for these two groups. The current results raise the concern that catastrophic health expenditure might be associated with health deterioration. However, it could lead to poverty in the long term, because excessive health expenditure increases the likelihood of an unmet healthcare need [31], eventually harming personal health. By way of comparison, Australia, like Korea, has implemented a universal healthcare system and it has shown persistent disparities in expenditure burden according to income [32]. Therefore, based on the results of this study, the burden of medical expenses for the second-lowest income bracket as a blind spot for the healthcare system should not be overlooked. Second, discussions should continue in order to address the occurrence of unmet healthcare needs and the gap in improved health levels. Finally, efforts should be made to resolve income and health inequality based on health insurance subscription types. Even in countries with universal healthcare, ongoing monitoring of healthcare spending is an essential part of assessing healthcare system performance.

This study has several limitations. First, information bias may have occurred because the main variables of this study, unmet healthcare needs and health status, were measured subjectively. However, it is common to use surveys on unmet healthcare needs as a subjective indicator to identify problems with access to healthcare. Second, endogenous factors could have affected unmet healthcare needs, catastrophic expenditure, and health status. Therefore, we created a mediation regression model that included the income levels and health insurance types that were most likely to affect both variables. However, future studies should include instrumental variables [33] and propensity score matching [34] to provide a more detailed analysis. In addition, recall bias in participants’ responses regarding their unmet healthcare needs and personal health status could have occurred because these factors were self-reported.

Despite these limitations, the results indicate that medical accessibility and health inequality could be exacerbated by income disparity and inequality in insurance coverage. Korea’s health insurance system underwent a bimodal reorganization into insurance coverage for employees and self-employed individuals, which contributes to the continued controversy over inequality between income brackets. To address this issue, the healthcare policy was revised to include a new insurance premium system that abolished estimated income, reduced the weight of automobiles and assets, and established the lowest insurance premium for self-employed individuals in July 2018. It is necessary to continue revising healthcare policies to enhance equality by integrating health insurance premiums for employees and self-employed individuals based on income alone.

## 5. Conclusions

Eliminating policy disparities is crucial to resolve healthcare inequality. It is imperative to understand inequity from the perspective of the poorest income groups and revise the policy paradigm to focus on disparity rather than average health. Efforts to improve insurance premiums for employees and self-employed individuals should be developed in line with the practical measures used in the MA selection criteria aimed at protecting those in the systematic blind spot (based on medical service use and health inequality). These measures are urgently needed to improve and provide a fair health insurance system in Korea.

## Figures and Tables

**Table 1:** General characteristics of the study population from 2011 to 2015.

		2011		2012		2013		2014		2015	
		*n*	%	*n*	%	*n*	%	*n*	%	*n*	%
Total		6713	100.0	6692	100.0	6576	100.0	6732	100.0	6724	100.0
Health insurance type and income level	High-income employee insured	943	16.4	916	16.2	910	17.0	909	16.8	1035	19.5
	Middle-income employee insured	2705	41.8	2781	42.9	2739	43.1	2815	44.5	2713	43.6
	Low-income employee insured	819	9.4	825	9.3	840	9.0	905	9.2	897	8.2
	High-income self-employed insured	256	4.1	250	4.2	239	4.2	250	4.1	284	4.4
	Middle-income self-employed insured	1264	19.3	1231	18.9	1186	18.4	1201	17.8	1148	17.1
	Low-income self-employed insured	455	5.6	434	5.3	407	5.0	418	4.9	403	4.3
	Medical aid	271	3.4	255	3.2	255	3.3	234	2.6	244	2.8
Sex	Male	2702	41.0	2742	42.0	2712	42.2	2757	42.3	2743	42.0
	Female	4011	59.0	3950	58.0	3864	57.8	3975	57.7	3981	58.0
Age (years)	18–34	882	18.6	807	17.3	705	16.4	637	15.7	554	14.8
	35–64	4046	62.6	4018	64.0	3904	65.0	4006	65.7	3958	66.6
	≥65	1785	18.8	1867	18.6	1967	18.6	2089	18.6	2212	18.6
Level of education	≥College	1728	30.2	1811	32.3	1797	33.3	1905	35.6	1926	37.3
	High school	2345	37.7	2377	38.4	2304	38.3	2329	37.4	2315	37.2
	Middle school	861	11.7	828	10.9	813	10.7	828	10.5	808	9.7
	≤Middle school	1779	20.3	1676	18.5	1662	17.7	1670	16.5	1675	15.8
Marital status	Married	5165	76.0	5169	76.5	5082	76.4	5179	76.0	5.107	74.7
	Divorce or widowed	904	11.0	891	10.6	903	10.5	968	10.6	1034	11.0
	Single	644	13.0	632	12.9	591	13.1	585	13.4	583	14.3
Job	Permanent employment	1216	21.6	1216	22.0	1214	22.7	1236	23.6	1249	24.9
	Temporary employment	1284	18.9	1284	20.5	1253	20.6	1330	20.7	1315	21.2
	Self-employment	1534	20.9	1534	20.8	1511	20.8	1560	20.7	1401	19.0
	Economically inactive population	2658	38.6	2658	36.7	2598	35.9	2606	34.9	2759	34.9
Chronic diseases	No	2192	38.3	2171	38.5	1912	36.1	1975	36.9	1954	37.6
	Yes	4521	61.7	4521	61.5	4664	63.9	4757	63.1	4770	62.4
Self-rated health	Good	5714	87.0	5726	86.9	5629	88.2	5579	86.4	5665	88.3
	Poor	999	13.0	1066	13.1	1153	11.8	1153	13.6	1059	11.7
Unmet health needs (economic reasons)	No	6365	95.1	6238	93.9	6135	94.2	6435	96.3	6391	96.4
	Yes	348	4.9	454	6.1	441	5.8	297	3.7	333	3.6
Catastrophic health expenditure	No	5646	86.4	5610	86.8	5475	86.6	5586	86.9	5543	87.3
	Yes	1067	13.6	1082	13.2	1101	13.4	1146	13.1	1181	12.7
		mean	SD	mean	SD	mean	SD	mean	SD	mean	SD
Number of family members		3.2	0.2	3.3	0.2	3.3	0.2	3.3	0.2	3.3	0.2
Proportion of economically active family members		0.5	0.1	0.5	0.1	0.5	0.1	0.5	0.1	0.5	0.1
% of family members having chronic diseases		0.5	0.1	0.5	0.1	0.5	0.1	0.5	0.1	0.5	0.1

Note: % is presented weighted %, values are presented as n (%); SD: standard deviation.

**Table 2:** General characteristics by self-reported health, unmet healthcare needs, and catastrophic health expenditure.

		Y: Self-Rated Health					M1: Unmet Healthcare Needs					M2: Catastrophic Health Expenditure				
		Good		Poor		*p*-Value	No		Yes		*p*-Value	No		Yes		*p*-Value
		*n*	%	*n*	%		*n*	%	*n*	%		*n*	%	*n*	%	
Total		28,213	87.3	5224	12.7		31,564	95.3	1873	4.7		27,860	86.8	5577	13.2	
Health insurance type and income level	High-income employee insured	4378	93.6	335	6.4	<0.001	4678	99.4	35	0.6	<0.001	4609	97.9	104	2.1	<0.001
	Middle-income employee insured	12,057	90.0	1696	10.0		13,266	96.9	487	3.1		12,051	89.5	1702	10.5	
	Low-income employee insured	3072	73.6	1214	26.4		3821	89.3	465	10.7		2278	55.9	2008	44.1	
	High-income self-employed insured	1170	92.2	109	7.8		1259	98.6	20	1.4		1255	98.3	24	1.7	
	Middle-income self-employed insured	5298	89.2	732	10.8		5740	95.1	290	4.9		5352	89.8	678	10.2	
	Low-income self-employed insured	1565	76.4	552	23.6		1787	85.8	330	14.2		1293	64.8	824	35.2	
	Medical aid	673	55.6	586	44.4		1013	79.8	246	20.2		1022	80.5	237	19.5	
Sex	Male	12,021	90.4	1635	9.6	<0.001	13,051	96.3	605	3.7	<0.001	11,425	87.3	2231	12.7	0.056
	Female	16,192	85.1	3589	14.9		18,513	94.6	1268	5.4		16,435	86.5	3346	13.5	
Age (years)	18–34	3421	95.6	164	4.4	<0.001	3513	98.1	72	1.9	<0.001	3282	91.9	303	8.1	<0.001
	35–64	17,749	89.9	2183	10.1		19,114	96.1	818	3.9		18,005	91.2	1927	8.8	
	≥65	7043	71.2	2877	28.8		8937	90.2	983	9.8		6573	67.3	3347	32.7	
Level of education	≥College	8611	94.4	556	5.6	<0.001	8975	98.1	192	1.9	<0.001	8537	93.9	630	6.1	<0.001
	High school	10,544	91.2	1126	8.8		11,255	96.6	415	3.7		10.303	89.9	1367	10.1	
	Middle school	3314	81.0	824	19.0		3863	93.1	275	6.9		3239	80.9	899	19.1	
	≤Middle school	5744	69.0	2718	31.0		7471	88.5	991	11.5		5781	70.3	2681	29.7	
Marital status	Married	22,061	88.2	3641	11.8	<0.001	24,510	96.1	1192	3.9	<0.001	21,535	87.3	4167	12.7	<0.001
	Divorce or widowed	3295	71.8	1405	28.2		4095	87.1	605	12.9		3610	79.3	1090	20.7	
	Single	2857	94.5	178	5.5		2959	97.7	76	2.3		2715	90.0	320	10.0	
Employment status	Permanent employment	5837	95.5	294	4.5	<0.001	6042	98.7	89	1.3	<0.001	5792	94.5	339	5.5	<0.001
	Temporary employment	5714	90.6	679	9.4		5996	94.6	397	5.4		5619	89.5	774	10.5	
	Self-employment	6372	87.0	1179	13.0		7194	95.8	357	4.2		6138	85.0	1413	15.0	
	Economically inactive	10,290	80.4	3072	19.6		12,332	93.3	1030	4.7		10,311	81.5	3051	18.6	
Chronic diseases	No	9851	96.7	353	3.3	<0.001	9965	97.9	239	2.1	<0.001	9474	93.3	730	6.7	<0.001
	Yes	18,362	81.7	4871	18.3		21,599	93.8	1634	6.2		18,386	83.0	4847	17.0	

Note: % is presented weighted %, values are presented as n (%).

**Table 3:** Effects of catastrophic health expenditure and unmet healthcare needs on health status according to national health insurance type and income level.

		Model 1-1			Model 1-2			Model 2			Model 3		
		X → M1			X → M2			X → Y			X, M1, M2 → Y		
		dx/dy	SE	*p*-Value	dx/dy	SE	*p*-Value	dx/dy	SE	*p*-Value	dx/dy	SE	*p*-Value
Health insurance type and income level	High-income employee insured												
	Middle-income employee insured	0.026	0.003	<0.001	0.099	0.005	<0.001	0.019	0.007	0.005	0.012	0.007	0.071
	Low-income employee insured	0.062	0.005	<0.001	0.270	0.010	<0.001	0.045	0.009	<0.001	0.022	0.009	0.015
	High-income self-employed insured	0.012	0.006	0.032	−0.017	0.006	0.008	−0.001	0.012	0.976	−0.001	0.012	0.955
	Middle-income self-employed insured	0.040	0.004	<0.001	0.835	0.006	<0.001	0.019	0.008	0.021	0.011	0.008	0.171
	Low-income self-employed insured	0.100	0.008	<0.001	0.254	0.012	<0.001	0.050	0.011	<0.001	0.024	0.010	0.023
	Medical aid	0.122	0.012	<0.001	0.081	0.011	<0.001	0.172	0.018	<0.001	0.156	0.017	<0.001
M1: Unmet healthcare needs	No												
	Yes										0.107	0.009	<0.001
M2: Catastrophic health expenditure	No												
	Yes										0.056	0.006	<0.001

Note. dx/dy = marginal effect, SE = standard error. Control variables: sex, age group, educational level, marital status, and employment status, number of family members, proportion of economically inactive family members, and proportion of family members with chronic diseases.

## Abbreviations

MA: medical aid; NHI: national health insurance.
